# Supplementary material for: An averaging model for analysis and interpretation of high-order genetic interactions
Source: PLoS One. 2024 Apr 10;19(4):e0299525. doi: 10.1371/journal.pone.0299525 (PMC11006166; doi:10.1371/journal.pone.0299525)
Supplement: S1 Text — (DOCX) [file pone.0299525.s001.docx]

Text S1. Derivation of the averaging model and non-distributivity of the data

*Laws of algebra*

An additive model of gene effects and interactions involves two operators: additive, “+”, and interactive, “:”. Different models can be derived if we assume different laws for these operations. Three types of laws define algebra involving two operators: commutative, associative, and distributive laws. The commutative laws are A + B = B + A and A:B = B:A. The associative laws are (A + B) + C = A + (B + C) and (A:B):C = A:(B:C). The distributive law is (A + B):C = A:C + B:C.

I assume the commutative laws for both “+” and “:” because a single quantitative phenotype cannot experimentally distinguish A + B from B + A or A:B from B:A. I also assume the associative law for “+” since without this assumption the general linear model framework cannot be used.

The associative law for “:” is also required for the general linear model framework (see below). However, as I show below, the impact of a violation of the associative law for “:” can be moderated using an averaging principle, in which the arithmetic mean of multiple different expressions for the same quantity is taken as the true value of the quantity. This moderation by the averaging principle is important in applications of the general linear model framework to multi-gene systems because we cannot generally assume that the associative law for “:” holds. For example, in the case of Fig. 1 in the main text, A:B = 0, and thus, (A:B):C = 0. However, B:C ≠ 0, so, A:(B:C) may not be 0 particularly when A:C ≠ 0. Thus, (A:B):C ≠ A:(B:C), i.e., a violation of the associative law, could happen.

In the following sections, I will show that the distributive law is required in the additive model. I will also show that a range-limiting nonlinearity of a system, such as a saturation response, would violate the distributive law. Such responses are common in biological systems. I will also show that there is a general linear model that allows violation of the distributive law under the assumption of the averaging principle. I call this model an averaging model.

*Derivation of the averaging model*

The part of the following discussion describing derivation of the NR model was modified from Text S1 in (2). In this section, for simplicity, the intercept value (i.e., the phenotype value for the most disrupted state) is subtracted from all the measured values so that the intercept value is 0.

According to the additive model, I assume *AB* = A + B + A:B … (1) as the starting point. In this case, the interaction is the deviation of the corresponding genotype from arithmetic addition of the 1-gene effects. Let’s extend this to a system consisting of three genes *A*, *B*, and *C*. The phenotype *ABC* can be considered as being expressed in three different ways: adding C to the genetic background of *AB*; adding A to the genetic background of *BC*; or adding B to the genetic background of *CA*.

By adding C to *AB*, the genotype *ABC* is expressed as:

*ABC* = *AB* + C + *AB*:C = (A + B + A:B) + C + *AB*:C = A + B + C + A:B + *AB*:C … (2)

If the distributive law is not assumed, (2) cannot be simplified.

If the distributive law is assumed, (2) can be simplified to:

*ABC* = A + B + C + A:B + (A + B + A:B):C = A + B + C + A:B + B:C + C:A + (A:B):C … (3)

Similarly, if the distributive law is not assumed:

By adding A to *BC*, *ABC* = A + B + C + B:C + *BC*:A … (4)

By adding B to *CA*, *ABC* = A + B + C + C:A + *CA*:B … (5)

If the distributive law is assumed:

By adding A to *BC*, *ABC* = A + B + C + A:B + B:C + C:A + A:(B:C) … (6)

By adding B to *CA*, *ABC* = A + B + C + A:B + B:C + C:A + B:(C:A) … (7)

Although the expressions are not the same, (3), (6), and (7) must be the same in a model to explain *ABC*. Therefore, for this model framework to work exactly, the associative law for the interaction operator “:” is necessary,

(A:B):C = A:(B:C) = B:(C:A) = A:B:C … (8)

If (8) is true, (3), (6), and (7) become the same expression:

*ABC* = A + B + C + A:B + B:C + C:A + A:B:C … (9)

(9) is the additive model for three genes. This can be extended to a system consisting of more genes. In summary, the additive model is a good description of a multi-gene system if the associative law for “:” and the distributive law are conserved.

However, as discussed above, the associative law cannot be generally assumed for the “:” operator in a multi-gene system. This contradiction about associativity indicates a failure of the general linear model as a general description of a multi-gene system. A compromise to maintain the general linear model framework is to define *ABC* as the arithmetic mean of (3), (6), and (7):

*ABC* = [{A + B + C + A:B + B:C + C:A + (A:B):C} + {A + B + C + A:B + B:C + C:A + A:(B:C)}

+ { A + B + C + A:B + B:C + C:A + B:(C:A)}] / 3

= A + B + C + A:B + B:C + C:A + {(A:B):C + A:(B:C) + B:(C:A)} / 3 … (10)

I call this practical approach to avoiding the contradiction in the general linear model by averaging all possible cases the averaging principle.

Since {(A:B):C + A:(B:C) + B:(C:A)} cannot be expressed by the lower order terms, A, B, C, A:B, B:C, and C:A, it is reasonable to define A:B:C = {(A:B):C + A:(B:C) + B:(C:A)} / 3 … (11). Then,

*ABC* = A + B + C + A:B + B:C + C:A + A:B:C … (9)

Thus, with the averaging principle, the additive model can conform to the assumption of no associativity in the interaction operator “:”. This can be extended to a system consisting of more genes. In summary, the additive model should be a reasonable description of a multi-gene system if the distributive law holds.

If the distributive law cannot be assumed, (2), (4), and (5) must still be the same to express *ABC*. Here again we observe a failure of the general linear model as a general description of a multi-gene system. I apply the averaging principle to (2), (4), and (5) to express *ABC*:

*ABC* = [{A + B + C + A:B + *AB*:C} + {A + B + C + B:C + *BC*:A} + {A + B + C + C:A + *CA*:B}] / 3

= A + B + C + (A:B + B:C + C:A) / 3 + (*AB*:C + *BC*:A + *CA*:B) / 3 … (12)

Since (*AB*:C + *BC*:A + *CA*:B) cannot be expressed by the lower order terms, A, B, C, A:B, B:C, and C:A, it is reasonable to define A;B;C = (*AB*:C + *BC*:A + *CA*:B) / 3 … (13). I use the semicolon “;” to distinguish this different definition of interaction from that of the interaction in the additive model and call “;” the averaging interaction operator and “:” the additive interaction operator.

*ABC* = A + B + C + (A:B + B:C + C:A) / 3 + A;B;C … (14)

Therefore, if the distributive law is not assumed, the average of the 2-gene additive interactions should be used to express the all wild-type allele state of *ABC*. In general, the rule that the terms in each order of the interactions (2-gene additive interactions, 3-gene averaging interactions, 4-gene averaging interactions, …) must be averaged can be derived by extending this to a system with more genes.

For example, with a system consisting of 4 genes, *A*, *B*, *C*, and *D*:

*ABCD* = A + B + C + D + (A:B + A:C + A:D + B:C + B:D + C:D) / 6 + (A;B;C + A;B;D + A;C;D + B;C;D) /4 + A;B;C;D … (15)

This is the NR model (7) (previously called the signaling allocation model (2)). Note that in the NR model, 2-gene interactions are additive interactions while 3- or higher-order interactions are averaging interactions.

The assumption of non-distributivity does not require any more changes in the model. However, the above derivation of the NR model began with an arbitrary definition of the 2-gene additive interaction, *AB* = A + B + A:B … (1), which is the reason the NR model is a mixture of additive and averaging interactions. The model would be more mathematically consistent if the averaging interaction definition were extended to 1-gene effect terms to make the 2-gene interactions averaging interactions as well, i.e., *AB* = (A + B) / 2 + A;B … (16). I demonstrate in Figs. 2 and 3 in the main text that (16) is indeed required for mathematical consistency of the model.

By applying (16):

*ABC* = (A + B + C) / 3 + (A;B + B;C + C;A) / 3 + A;B;C … (17)

*ABCD* = (A + B + C + D) / 4 + (A;B + A;C + A;D + B;C + B;D + C;D) / 6 + (A;B;C + A;B;D + A;C;D + B;C;D) /4 + A;B;C;D … (18)

Now the rule is that the terms in each order of the interactions, including 1-gene effects (the first order), must be averaged. (17) and (18) are equivalents of:

*ABC* = (*AB* + *BC* + *CA*) / 3 + A;B;C … (19)

A;B;C = *ABC* - (*AB* + *BC* + *CA*) / 3 … (19)’ (variated from (19))

*ABCD* = (*ABC* + *ABD* + *ACD* + *BCD*) /4 + A;B;C;D … (20)

A;B;C;D = *ABCD* - (*ABC* + *ABD* + *ACD* + *BCD*) /4 …(20)’ (variated from (20))

From (19)’ and (20)’, the highest order averaging interaction is defined as the deviation of the corresponding genotype from the average of all genotypes with one gene fewer. This definition of the averaging interaction is highly interpretable. I call this extended model with all averaging interactions an averaging model. With the definitions of the averaging interactions of different orders in (16), (19), and (20), it is clear that the averaging model does not require the distributive law because these definitions do not include any terms that could be affected by whether the distributive law holds or not.

Note that the mean estimates from the additive model, NR model, averaging model, and 1-way ANOVA for all genotypes are just different ways to linearly decompose the phenotype values (when the full model terms are kept). Thus, when the models are fit to actual data with replication, all these models yield the same fitted and residual values. The numbers of estimated values are the same, i.e., the models have the same residual degree of freedom. Therefore, I sometimes use only the mean estimates of the models for my arguments. The coefficient matrices to solve the linear equations for the means in the three models using the genotype mean values in a 3-gene system are shown in Fig. TS1.1.


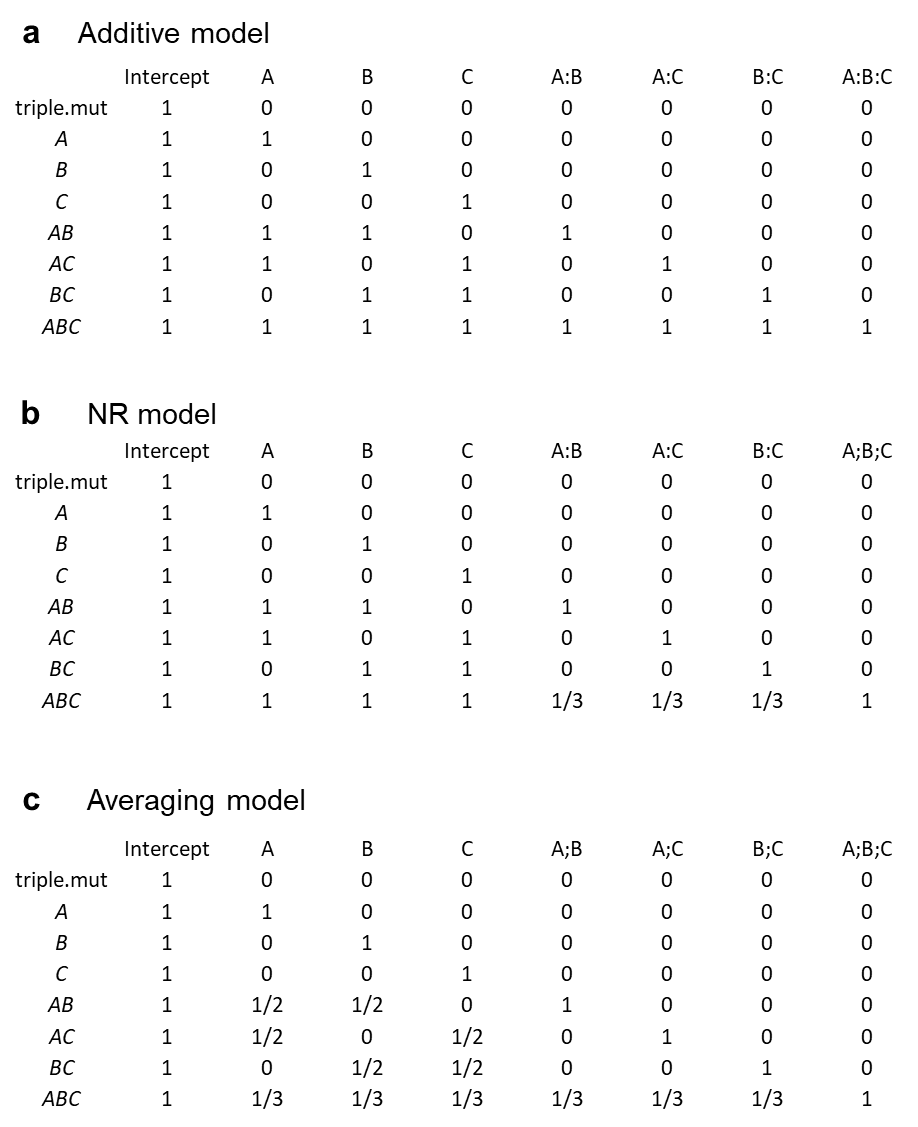


Fig. TS1.1. Matrices for the linear equations to obtain model coefficients from the genotype values in a 3-gene system for (a) additive, (b) NR, and (c) averaging models. The rows are genotypes and the columns are model variables. “:” and “;” indicate the additive and averaging interactions, respectively.

*Violation of the distributive law is prevalent in multi-gene systems*

The averaging model does not assume the distributive law. Do we really need to consider non-distributivity in a biological system? Let’s consider a simple 3-gene system, in which nA and nB are input nodes and nC is the output node (Fig. TS1.2). Mechanistically, signals from nA and nB are first summed, and then modulated by a nonlinear function $f_{1}$ before the signal is output from nC. Thus,

(A + B):C = nC = $f_{1}$(nA + nB) … (21)

A:C + B:C = $f_{1}$ (nA) + $f_{1}$ (nB) … (22)

Therefore, if the distributive law holds,

$f_{1}$(nA + nB) = $f_{1}$ (nA) + $f_{1}$ (nB) … (23)

Let’s make $f_{1}$ a Michaelis-Menten function for a saturating response (Fig. TS1.2b):

$f_{1}\left( x \right)=\frac{10}{1+\frac{7}{x}}$ … (24)

When nA = 5, nB = 2,

$f_{1}$(nA + nB) = $f_{1}(5+2)$ = 5

$f_{1}$ (nA) + $f_{1}$ (nB) = $f_{1}\left( 5 \right)+f_{1}(2)$ = 4.16… + 2.22… = 6.38…

Thus, $f_{1}$(nA + nB) $\neq f_{1}$ (nA) + $f_{1}$ (nB) … (25)

and the distributive law is violated.

Generally, nonlinearity in a system leads to violation of the distributive law.

Fig. TS1.2. Non-linearity in a system violates the distributive law. (a) a 3-gene system, in which signals from nA and nB feed into nC. The output of nC is defined as $f_{1}$(nA + nB). (b) When $f_{1}\left( x \right)=\frac{10}{1+\frac{7}{x}}$ , the input-output relationships at nC are shown. If the input is nA, the output is expressed as A:C in the additive model. This plot clearly shows that (A + B):C ≠ A:C + B:C (Y-axis values in orange and green, respectively), a violation of the distributive law.


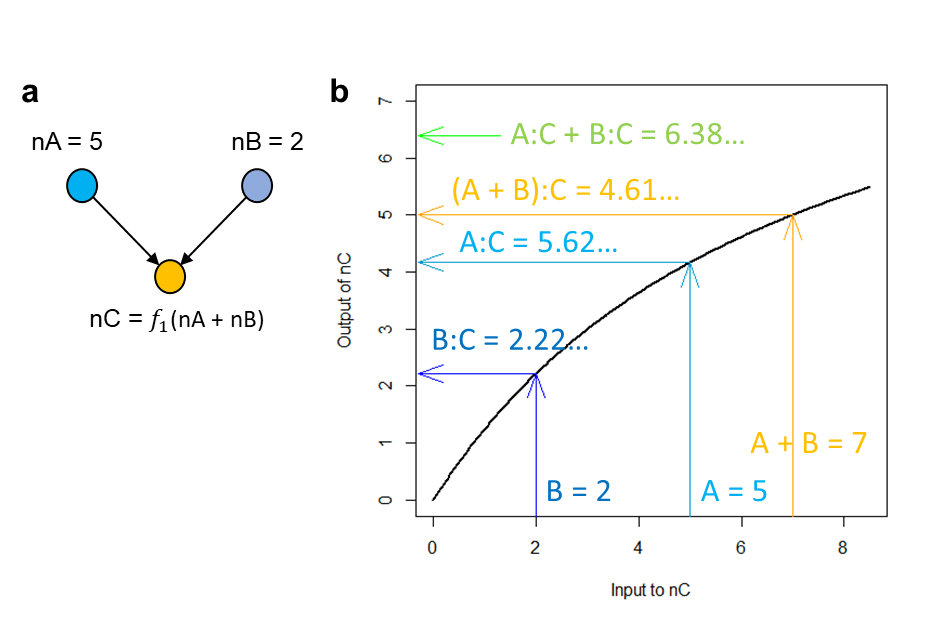


A saturating response limits the output range. Without nonlinearity, the range of the system output is not limited, and this is the condition the additive model requires. Thus, the additive model generally cannot be used in a system consisting of multiple genes (when the model includes 3 -or-more-gene interactions, strictly speaking) when the phenotype value range is limited compared to the ranges of the gene effects and interactions.

**References**

1. K. Tsuda, M. Sato, T. Stoddard, J. Glazebrook, F. Katagiri, Network properties of robust immunity in plants. *PLoS Genet* **5**, e1000772 (2009).

2. F. Katagiri, Network Reconstitution for Quantitative Subnetwork Interaction Analysis. *Methods Mol Biol* **1578**, 223-231 (2017).
