# Supplementary material for: An averaging model for analysis and interpretation of high-order genetic interactions
Source: PLoS One. 2024 Apr 10;19(4):e0299525. doi: 10.1371/journal.pone.0299525 (PMC11006166; doi:10.1371/journal.pone.0299525)
Supplement: S2 Text — (DOCX) [file pone.0299525.s002.docx]

Text S2. Averaging and additive models without interaction are two extreme approximations of the behavior of a two-input chemical signaling system

We consider a hypothetical system with two converging input signals. One input is signal molecule A, the other input is signal molecule B, and the output is signal molecule C (Fig. S2.1).


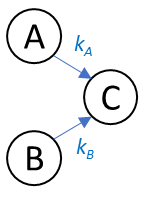


Fig. S2.1. The structure of a two-input system.

The system is modeled assuming an equilibrium among the three signal molecules. It can also be considered as a steady state of a dynamical system, which is more realistic for a signaling system. The system can be considered as a system of chemical reactions (e.g., A + X → C and B + Y → C), in which other molecules in the reactions (like X and Y) are not limiting (i.e., [X], [Y] >> [A], [B], [C]). We can only observe the final output of [C], the concentration of C, under the conditions of presence or absence of A and B. We cannot observe [A] and [B].

In the system, the sum of [A] and the fraction of [C] that derived from [A] is denoted as [A]_0_, and the sum of [B] and the fraction of [C] that derived from [B] is denoted as [B]_0_. When the system lacks B (genotype *Ab*), [C]_A_, the concentration of C specific to this condition is expressed with [A]_A_, the concentration of A specific to this condition, and the conversion constant *k_A_* as [C]_A_ = *k_A_*[A]_A_. Since [C]_A_ = [A]_0_ – [A]_A_, by eliminating [A]_A_,

[C]_A_ = { *k_A_* / ( *k_A_* + 1) } [A]_0_ … (1)

Similarly, when the system lacks A (genotype *aB*), with the conversion constant *k_B_*,

[C]_B_ = { *k_B_* / ( *k_B_* + 1) } [B]_0_ … (2)

When the system has both A and B (genotype *AB*), with [A]_AB_ and [B]_AB_, the concentration of A and B specific to this condition, [C]_AB_, the concentration of C specific to this condition, can be expressed as:

[C]_AB_ = *k_A_*[A]_AB_ = *k_B_*[B]_AB_ … (3)

Also, [C]_AB_ = ([A]_0_ – [A]_AB_) + ([B]_0_ – [B]_AB_) … (4)

By eliminating [A]_AB_ and [B]_AB_ from (3) and (4),

[C]_AB_ = { 1 / (1 / *k_A_* + 1/ *k_B_* + 1) } ([A]_0_ + [B]_0_) … (5)

We now consider the situations with different *k_A_* and *k_B_* values.

I. When 0 < *k_A_*, *k_B_* << 1,

(1), (2), and (5) can be approximated as:

[C]_A_ ≈ *k_A_*[A]_0_ … (6)

[C]_B_ ≈ *k_B_*[B]_0_ … (7)

[C]_AB_ ≈ { 1 / (1 / *k_A_* + 1 / *k_B_*) } ([A]_0_ + [B]_0_) … (8)

*k_A_* and [A]_0_ cannot be distinguished as we only know [C]_A_ as the phenotype, and similarly, *k_B_* and [B]_0_ cannot be distinguished as we only know [C]_B_ as the phenotype. Thus, we assume *k* ≈ *k_A_* ≈ *k_B_* to remove unnecessary complexity from the model. Then,

[C]_A_ ≈ *k*[A]_0_ … (6)’

[C]_B_ ≈ *k*[B]_0_ … (7)’

[C]_AB_ ≈ *k* ([A]_0_ + [B]_0_) / 2 … (8)’

From (6)’, (7)’, and (8)’,

[C]_AB_ ≈ ( [C]_A_ + [C]_B_ ) / 2 … (9)

Therefore, under the conditions of 0 < *k_A_*, *k_B_* << 1 (meaning [A],[B] >> [C]), the averaging model without interaction describes the system well.

II. When *k_A_*, *k_B_* >> 1,

(1), (2), and (5) can be approximated as:

[C]_A_ ≈ [A]_0_ … (10)

[C]_B_ ≈ [B]_0_ … (11)

[C]_AB_ ≈ [A]_0_ + [B]_0_ … (12)

From (10), (11), and (12),

[C]_AB_ ≈ [C]_A_ + [C]_B_ … (13)

Therefore, under the conditions of *k_A_*, *k_B_* >> 1 (meaning [A],[B] << [C]), the additive model without interaction describes the system well.

The discussion in this supplemental text demonstrates that whether the averaging model or the additive model describes the no interaction state better in a two-input system depends on the system. Since we can only observe the output of the system, [C], (but not [A] or [B]), we cannot determine which of the averaging and additive models would be better for a particular system.
