# Supplementary material for: An averaging model for analysis and interpretation of high-order genetic interactions
Source: PLoS One. 2024 Apr 10;19(4):e0299525. doi: 10.1371/journal.pone.0299525 (PMC11006166; doi:10.1371/journal.pone.0299525)
Supplement: S1 File — Supplemental Datasets are available from https://github.com/fumikatagiri/Averaging_Model. (DOCX) [file pone.0299525.s003.docx]

**SUPPLEMENTAL DATASETS**

Supplemental Datasets are available from <https://github.com/fumikatagiri/Averaging_Model>.
